# Effects of oxybutynin transdermal system on health-related quality of life and safety in men with overactive bladder and prostate conditions

**DOI:** 10.1111/j.1742-1241.2007.01625.x

**Published:** 2008-01

**Authors:** D R Staskin, M T Rosenberg, N V Dahl, P V Polishuk, N R Zinner

**Affiliations:** 1 Weill Medical College of Cornell University New York, NY, USA; 2 Mid-Michigan Health Centers Jackson, MI, USA; 3 Watson Laboratories Morristown, NJ, USA; 4 Palomar Urology Escondido, CA, USA; 5 Western Clinical Research Torrance, CA, USA

## Abstract

**Aims:**

Overactive bladder (OAB) is common in men and may exist concomitantly with benign prostatic hyperplasia (BPH) and obstruction. We present a subanalysis of results from men with OAB in a 6-month, open-label study of treatment with the oxybutynin transdermal system (OXY-TDS). Broad entry criteria were incorporated to yield a clinically representative population.

**Methods:**

All participants received OXY-TDS 3.9 mg/day. Effectiveness was assessed by changes in scores on validated questionnaires, which included the single-item Patient Perception of Bladder Condition (PPBC), the King's Health Questionnaire (KHQ) and the Beck Depression Inventory-II (BDI-II).

**Results:**

The proportion of men (*n* = 369; mean age = 69.6 years) who reported that their bladder condition caused moderate, severe or many severe problems (PPBC ≥ 4) improved from 77.3% at baseline to 38.1–53.6% in subsequent months. Mean KHQ scores decreased significantly (p ≤ 0.0196) from baseline to study end in eight of 10 domains, indicating improved health-related quality of life. The proportion of men with BDI-II score > 12 (associated with a diagnosis of depression) decreased from 23.9% to 17.9% (p = 0.0055). Men with a history of ‘prostate problems’ or use of ‘BPH medication’ (32.2%) had KHQ domain changes that were similar (p ≥ 0.1016) to those of other men. Most men (76.2%) reported no treatment-related adverse events; two men (0.5%) experienced symptoms of mild urinary retention, but neither required catheterisation.

**Conclusions:**

Oxybutynin transdermal system treatment of men with OAB was effective and well tolerated, regardless of history of prostate condition.

**What's known**

- Combined treatment of men with and without BPH is an evolving paradigm.

**What's new**

- This article contributes significant safety data, from the largest study to date, in a community use situation, where anticholinergics are commonly used.
- The study provides significant quality of life benefit data in a large population.
- The community usage design did not employ inclusion or exclusion criteria that would restrict the primary care physician from administrating the medication in a ‘real life’ setting.

## Introduction

The overall prevalence of overactive bladder (OAB) in the USA is similar in men (16.1%) and women (16.9%) but increases with age and may become higher in men than in women after 75 years of age (1,2). Benign prostatic hyperplasia and obstruction (BPH and BPO) are conditions that may coexist in men and potentially complicate OAB treatment, and that are more common in older age groups (3). OAB and BPH are known to impair health-related quality of life (HRQoL) and to increase symptoms of depression (2,4–6). Studies of OAB treatments have focused primarily on patients with urinary incontinence (UI), most of whom are women (2). As a result, men have been under-represented in studies of treatment for OAB, in which they typically constitute < 15% of the population.

It is well established that orally administered antimuscarinic drugs are effective in reducing symptoms and improving HRQoL in patients with OAB, including those who are male (7–10). However, patients with OAB often are prescribed medications intended to treat BPH (11). Furthermore, antimuscarinic drugs are rarely prescribed together with BPH medications, perhaps because of concern that their anticholinergic effects may exacerbate obstructive symptoms (11). Patients often discontinue therapy with orally administered antimuscarinic medications because of systemic anticholinergic effects, such as dry mouth (12,13).

Guidelines from the 6th International Consultation on New Developments in Prostate Cancer and Prostate Diseases recommend antimuscarinic therapy for some older men with lower urinary tract symptoms (LUTS) (14). The guidelines on basic management suggest fluid restriction, lifestyle modification and bladder training (14). For patients with persistent, bothersome LUTS after basic management, suggested approaches for specialised management of OAB and bladder outlet obstruction (BOO) include both pharmacological therapy and surgical treatment (14). The direction of medical therapy depends on symptoms and the results of specific tests. If patients have mixed OAB and BOO, it is recommended that they be treated with antimuscarinics and α_1A_-adrenergic receptor antagonists (α-blockers) (14). When BOO is the predominant condition, α-blockers are recommended if patients have a small gland or a low (< 1.5 ng/ml) prostate-specific antigen (PSA) level, but both α-blockers and 5α-reductase inhibitors (5-ARI) should be prescribed if patients have an enlarged gland or a higher (> 1.5 ng/ml) PSA level (14).

Several studies have suggested that antimuscarinics and α-blockers can be used concurrently in men with OAB and BOO or BPH. A randomised, controlled efficacy and safety study of extended-release tolterodine (TOL-ER) and tamsulosin treatment in men (*N* = 879) with LUTS, including OAB, concluded that combination therapy was more efficacious than monotherapy and had similar tolerability (15). Lee et al. (16) studied men (*N* = 144) with LUTS who presented consecutively to a single tertiary care centre. Patients were classified into two groups: one in which patients had BOO alone (76/144; 53%), and a second group in which patients had BOO plus OAB (68/144; 47%) (16). OAB was defined as involuntary detrusor contractions at ≥ 10 cm H_2_O (16). Improvement was defined as at least a three-point reduction in International Prostate Symptom Score (IPSS) (16). In the group with BOO plus OAB, only 35% (24/68) of men treated with doxazosin alone reported improvement in symptoms at the end of the initial 3-month period (16). The response rate in this group increased to 82% (56/68) 2 months after 2 mg tolterodine twice daily was added to doxazosin therapy (16).

An alternative to oral administration for antimuscarinic medication, if its use resulted in a lower incidence of dry mouth and other anticholinergic adverse effects, might be attractive to patients. The oxybutynin transdermal system (OXY-TDS; Oxytrol®, Watson Laboratories, Morristown, NJ) has been shown to be as effective as orally administered oxybutynin immediate release (OXY-IR) and TOL-ER in reducing symptoms of OAB (17,18), but OXY-TDS is associated with a lower rate of dry mouth (17,18). Inhibition of saliva production during treatment with oxybutynin, a commonly used antimuscarinic OAB drug, appears to be correlated with plasma concentrations of the metabolite *N*-desethyloxybutynin (19). Delivery of oxybutynin directly through the skin with OXY-TDS avoids the first-pass hepatic metabolism that occurs with orally administered oxybutynin (19). As a result, the incidence of anticholinergic adverse events with OXY-TDS therapy is similar to that with placebo transdermal treatment (18,20,21).

The Multicenter Assessment of TRansdermal therapy In overactive bladder with oXybutynin (MATRIX) study was undertaken to examine the effect of OXY-TDS therapy on HRQoL and safety in a large, diverse population. Here, we present a planned analysis of results from the cohort of men who participated in MATRIX, along with a *post hoc* analysis of men with and without pre-existing prostate conditions, to examine the safety and effectiveness of OXY-TDS treatment for patients with OAB.

## Methods

### Participant selection criteria

The MATRIX (study OXY0402; registered at ClinicalTrials.gov as NCT00224146) was an open-label, prospective, randomised, multicentre cohort study of community-based adults with OAB. The study design and main results, as well as data on sexual function, have been published elsewhere (22,23). Inclusion criteria consisted of one or more symptoms of OAB, as described on the OXY-TDS label (urge UI, urgency or frequency), age 18 years or older, and the ability to complete questionnaires without assistance. Exclusion criteria included any of the following current conditions: uncontrolled narrow-angle glaucoma, urinary retention or a treatable condition besides OAB that could cause UI or urgency (urinary tract infection, bladder stone, bladder tumour, prostatitis and prostate cancer). Participants could not have experienced hypersensitivity to oxybutynin or other components of OXY-TDS and could not have received prior treatment with OXY-TDS; they also could not be residents in a nursing home or a long-term care facility.

No exclusion criteria were based on maximum age, comorbid conditions besides those already described, or use of concurrent medications except other OAB treatments (or any investigational product within the previous 30 days). Participants could be enrolled despite current use of BPH medication or a history of prostate cancer. Written, informed consent was obtained from each individual before enrolment. MATRIX was conducted in accordance with the Declaration of Helsinki and Good Clinical Practice guidelines. The study protocol and procedures were approved by a central institutional review board (Independent Review Consulting, Corte Madera, CA) or, at a small number of study centres, by site-specific institutional review boards.

### Treatment

All participants received OXY-TDS at the US Food and Drug Administration-approved dosage of 3.9 mg/day (two patches per week) for up to 6 months. Study centres were randomised 1 : 1 within each investigator specialty (Primary Medicine, Obstetrics–Gynecology, Urology, Geriatrics or other) to provide to participants standard instructions for OXY-TDS or standard instructions plus a packet of enhanced OAB educational materials. Assistance with applying the first patch was provided at the enrolling centre; for subsequent applications, participants were instructed to rotate the position of patch application between the abdomen, hip and buttock. Participants returned to the clinic for visits after 1, 3 and 6 months of treatment.

### Participant assessment of treatment effectiveness

Participants rated their global OAB severity with use of the Patient Perception of Bladder Condition (PPBC), a validated, single-item questionnaire (24). Participants rated the difficulties caused by their bladder condition on the following six-point scale: ‘no problems at all’ (one), ‘very minor problems’ (two), ‘minor problems’ (three), ‘moderate problems’ (four), ‘severe problems’ (five) and ‘many severe problems’ (six).

Patient Perception of Bladder Condition assessments were made at the baseline clinic visit and were included during subsequent monthly scripted computer-assisted telephone interviews (CATI).

### Assessment of health-related quality of life impairment

Data on impairment of HRQoL were acquired through use of the King's Health Questionnaire® (KHQ), which was administered in the clinic at baseline, 3 months and 6 months. For participants who withdrew from the study before 6 months, the KHQ and other questionnaires were administered at the time of discontinuation. The KHQ is a 27-item, validated instrument that is used to assess symptoms and impairment of HRQoL in individuals with OAB or other lower urinary tract conditions (25). Although the KHQ was originally designed for use with women, it was subsequently validated for use in men (26). Results are converted to scores that pertain to 10 domains, with a score of zero corresponding to the least HRQoL impairment and a score of 100 signifying the worst HRQoL impairment. The minimal clinically important decrease (improvement) for most domain scores is considered to be five points; a decrease of three points is considered important for the domains of General Health Perception and Symptom Severity (27).

### Assessment of symptoms of depression

Information related to depression was obtained with the Beck Depression Inventory-II® (BDI-II), a validated 21-item questionnaire that was used to assess the existence and severity of symptoms associated with depression that occurred over the preceding 2 weeks (28). Each questionnaire item contains a list of statements about a particular symptom of depression, arranged in order of increasing severity; these items are consistent with the *Diagnostic and Statistical Manual of Mental Health Disorders, Fourth Edition*. BDI-II summary scores can range from zero (least depression) to 63 (greatest depression); a score > 12 is associated with a clinical diagnosis of depression.

### Participant-reported satisfaction with treatment

Participants were queried about their perceptions of the OXY-TDS patch during the monthly, scripted CATI sessions. Those who had been treated previously for OAB were asked to compare characteristics of OXY-TDS with those of their prior therapy.

### Assessment of safety

Safety was assessed by reports of adverse events recorded at postbaseline clinic visits (at 1, 3 and 6 months), or as they were reported at any time during the study. Investigators categorised adverse events by severity (mild, moderate or severe) and by possible causal relationship (related or not related) to treatment.

### Statistical analysis

To provide sufficient statistical power for analyses of patient subgroups, the target population size was set at approximately 2500 participants. Analyses of effectiveness were performed with the intent-to-treat population, which comprised all participants who received at least two doses of OXY-TDS and underwent at least one postbaseline assessment. The primary effectiveness end-point in MATRIX was mean change in KHQ domain scores from baseline to study end; analyses of select KHQ item responses also are presented here. A *post hoc* analysis of KHQ results was conducted for men with and without pre-existing prostate problems. Secondary effectiveness end-points included self-reported changes in global OAB condition (PPBC score), mean change from baseline to study end in BDI-II summary score, and participant perceptions of the OXY-TDS patch. Safety was analysed for all participants who received at least one dose of OXY-TDS.

The significance of differences between groups in continuous variables was determined by analysis of co-variance (ANCOVA). For comparison of baseline differences, the ANCOVA model included controls for investigator specialty. For end of study differences, controls included investigator specialty and baseline value, their interaction, and patient age. A chi-squared test was used to compare baseline differences in proportions of categorical variables. The Cochran–Mantel–Haenszel (CMH) test was used to compare baseline differences in proportions of ordered categorical variables. A one-sample, two-tailed *t*-test was used to assess the significance of mean changes in continuous variables (KHQ domain scores and BDI-II summary score) within groups from baseline to study end. The kappa test of symmetry was used to assess the significance of changes from baseline in PPBC scores at 3 and 6 months among participants with postbaseline values. The kappa test of symmetry was also used to determine the significance of changes in individual KHQ questionnaire response items from baseline to study end; for display, response items were grouped as ‘improved,’‘stayed the same’ or ‘worsened’. McNemar's test was used to calculate p-values for frequencies of paired data. CATI results were analysed with descriptive statistics.

## Results

### Participants

A total of 2888 individuals were enrolled at 327 study centres distributed throughout the continental USA (22). The analysis population consisted of all participants who were treated with OXY-TDS (*N* = 2878) (22). A substantial number of men (*n* = 369; 12.8%) participated, although most participants were female (*n* = 2508; 87.2%) (22). Demographic and baseline disease characteristics of male participants are presented in Table 1. Men in MATRIX were significantly (p < 0.0001; ANCOVA) older than female study participants (mean, 69.6 vs. 61.4 years); 71.3% of men (*n* = 263) were 65 years or older. Most men were Caucasian (*n* = 309; 83.7%), and about one-quarter were employed (*n* = 87; 23.6%). OAB symptoms had started ≥ 4 years ago in 39.3% of men. A majority of men (*n* = 207; 56.4%) had been treated at some time for OAB; the most common prior treatments were TOL-ER (*n* = 104; 28.2%), extended-release oxybutynin (*n* = 85; 23.0%), immediate-release tolterodine (TOL-IR; *n* = 46; 12.5%) and OXY-IR (*n* = 42; 11.4%). Cardiovascular disease was the most common type of comorbidity (*n* = 245; 66.4%), and male participants most commonly used concomitant medications related to treatment of this condition: 147 (39.8%) lipid-modifying agents, 139 (37.7%) anti-thrombotic agents and 131 (35.5%) agents that act on the renin–angiotensin system. Nearly all men (*n* = 320; 86.7%) were taking at least one concomitant medication.

**Table 1:** Demographic and baseline characteristics of male participants

Characteristics	Men (*n* = 369)
**Age, years**	
Mean (standard deviation)	69.6 (13.1)
Median (range)	72.0 (20–96)
**Race**, ***n*** (%)	
White	309 (83.7)
Black	36 (9.8)
Asian	10 (2.7)
Hispanic	9 (2.4)
Others	5 (1.4)
Employment status*, ***n*** (%)	
Employed†	87 (23.6)
Not employed‡	281 (76.4)
**Comorbid conditions by body system**, ***n*** (%)	
Cardiovascular	245 (66.4)
Musculoskeletal	169 (45.8)
Gastrointestinal	138 (37.4)
Endocrine	116 (31.4)
Neurological/psychiatric	113 (30.6)
Renal	82 (22.2)
Respiratory	74 (20.1)
Dermatological	50 (13.6)
Haematological/lymphatic	24 (6.5)
Others	204 (55.3)
**Number of concurrent medications**	
Mean (standard deviation)	5.8 (4.7)
Median (range)	5 (0–21)

* One participant did not respond.

† Working part time or full time, with or without pay.

‡ Unemployed, disabled, retired or other.

About one-third of men (119/369; 32.2%) had pre-existing prostate problems; of these, 67 were concurrently receiving BPH medication (α-blocker and/or 5-ARI). Men with prostate problems were older than other male participants (mean age, 73.0 vs. 68.0 years; p = 0.0010; ANCOVA) but were similar in ethnicity (p = 0.4122; chi-squared test) and employment status (p = 0.4783; chi-squared test). The proportions of men with and without prostate problems were well balanced for diuretic use at baseline (26.1% vs. 20.4%; p = 0.2223; chi-squared test). Time since onset of OAB symptoms was similar (p = 0.3310; CMH test) in the two groups. A higher proportion of men with prostate problems (73 of 118 respondents; 61.9%) than without (134 of 249 respondents; 53.8%) had been treated previously for OAB; men with prostate problems (29/119; 24.4%) were more likely than other men (46/250; 18.4%) to have taken multiple OAB medications previously. More men with prostate problems than men without prostate problems had discontinued prior OAB treatments because of ineffectiveness (61.2% vs. 56.3% respectively) and fewer because of compliance (2.6% vs. 8.9%), but other reasons for discontinuation of prior OAB therapies were given with similar frequency (‘side effects’, 21.6% vs. 19.8%; other, 12.9% vs. 12.5%; unknown, 1.7% vs. 2.6%).

### Participant-reported perceptions of bladder condition

**Figure 1:**
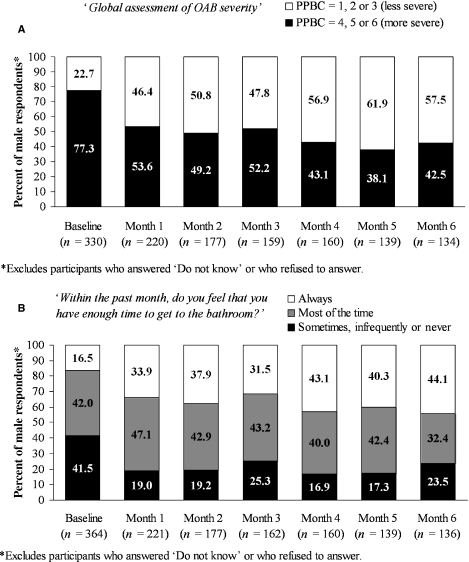
Participant-reported measures of bladder condition; percentages are calculated on the basis of the total number of respondents at each evaluation. (A) Percentage of respondents who rated their global overactive bladder (OAB) severity as 1, 2 or 3 (no problems at all, very minor problems or some very minor problems) or 4, 5 or 6 (moderate problems, severe problems or many severe problems) with the Patient Perception of Bladder Condition (PPBC) questionnaire. (B) Percentage of respondents who reported feeling that they had enough time to get to the bathroom during the past month

At the baseline clinic visit, most male participants who responded to the PPBC (255/330; 77.3%) reported a global OAB severity score of ≥ 4 (Figure 1A). This percentage decreased sharply after 1 month of OXY-TDS treatment and continued on a declining trend for the remainder of the study (Figure 1A). At baseline, 16.5% of male respondents (60/364) felt that they ‘always’ had enough time to get to the bathroom (Figure 1B). After 1 month, the proportion who ‘always’ had enough time to get the bathroom had approximately doubled to 33.9% (75/221); the proportion was stable or increased slightly over the next 5 months (Figure 1B).

### KHQ response items and health-related quality of life

**Figure 2:**
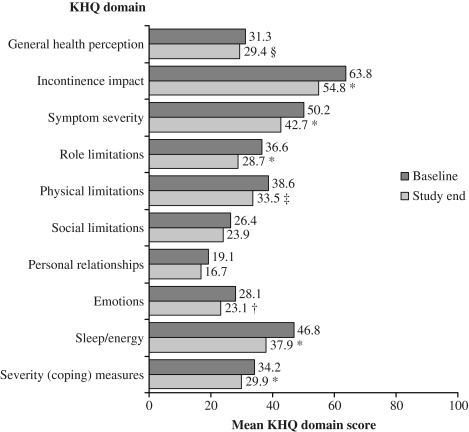
Health-related quality of life (HRQoL) of male participants at baseline and at study end, assessed as mean King's Health Questionnaire (KHQ) domain scores. *p < 0.0001, †p < 0.001, ‡p < 0.01, §p ≤ 0.0196; one-sample *t*-test for significance of difference from zero for change in baseline value; other changes were not statistically significant (p ≥ 0.1033)

At baseline, men in the study showed impairment of HRQoL in all 10 domains of the KHQ (Figure 2). The most impaired domains (highest scores) were Incontinence Impact, Symptom Severity, Sleep/Energy and Physical Limitations (Figure 2). By study end, scores had improved in all KHQ domains, and changes were statistically significant (p ≤ 0.0196; *t*-test) in eight of 10 domains (Figure 2). Changes were clinically meaningful in four of these eight domains: Incontinence Impact, Symptom Severity, Sleep/Energy and Role Limitations. The greatest absolute improvement was noted in the Incontinence Impact domain (mean change of −8.9 points), and the greatest relative improvement was seen in the Role Limitations domain (mean change of −21.0%).

**Figure 3:**
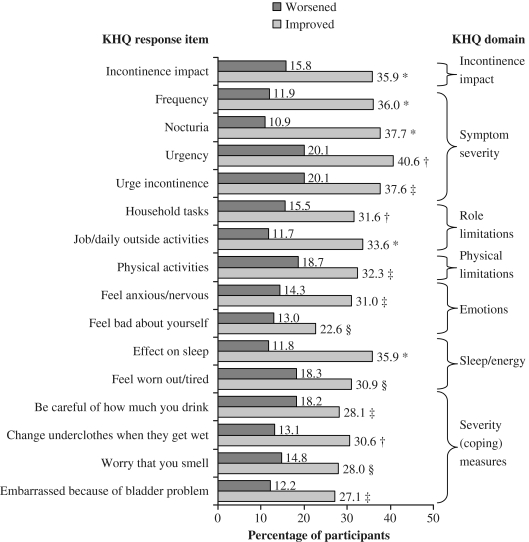
Percentage of male participants who reported improvement or worsening, from baseline to study end, in symptoms described in individual King's Health Questionnaire (KHQ) response items. Participants who reported that their symptoms ‘stayed the same’ are not depicted. Only responses for which statistically significant differences were noted are displayed. *p < 0.0001, †p < 0.001, ‡p < 0.01, §p ≤ 0.0488; kappa test of symmetry; other comparisons were not significantly different (p ≥ 0.1781)

For 16 KHQ items, measured at baseline and study end, significantly more male participants reported improvement of symptoms than the number who reported worsening of symptoms (Figure 3). This was most evident in response items from the domains of Incontinence Impact, Symptom Severity, Role Limitations, Sleep/Energy and Severity (Coping) Measures (Figure 3). For the remaining KHQ items, no significant difference was noted in the number of participants who reported improvement or worsening of symptoms.

### Health-related quality of life in men with and without pre-existing prostate problems

**Figure 4:**
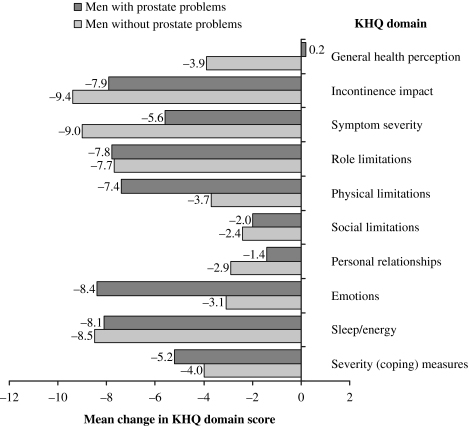
Improvement in health-related quality of life (HRQoL) in men with and without pre-existing prostate problems, assessed as mean changes in King's Health Questionnaire (KHQ) domain scores from baseline to study end. All changes in score were similar between the two groups (p ≥ 0.1016); analysis of co-variance (ANCOVA)

Men with and without pre-existing prostate problems had similar (p ≥ 0.1575; ANCOVA) baseline scores in all 10 KHQ domains. HRQoL improved in both groups from baseline to study end, as was shown by mean decreases in nine of 10 KHQ domain scores (Figure 4); changes were similar (p ≥ 0.1016; ANCOVA) in all 10 domains for both groups.

### Symptoms of depression

The mean BDI-II summary score at baseline for men in the study was 8.5 points; the median score was 7.0. By study end, the mean BDI-II summary score had improved significantly (p < 0.0001; *t*-test) to 7.1 – a decrease of 16.5%. Median BDI-II summary score decreased to 6.0 by study end. Among participants with postbaseline data, the proportion of men with a BDI-II summary score > 12, which is associated with a diagnosis of clinical depression, decreased significantly, from 23.9% (72/301) at baseline to 17.9% (54/301) at study end (p = 0.0055; McNemar's test).

### Participant-reported satisfaction with treatment

**Figure 5:**
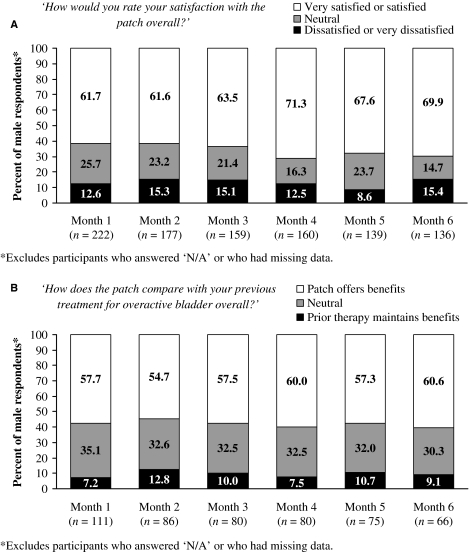
Participant-reported satisfaction with oxybutynin transdermal system (OXY-TDS) treatment; percentages are calculated on the basis of the total number of respondents at each evaluation. (A) Overall satisfaction in the total population of male participants. (B) Overall satisfaction in male participants who had been treated before for overactive bladder (OAB)

Most male participants (61.6–71.3%) were ‘very satisfied’ or ‘satisfied’ with OXY-TDS throughout the study (Figure 5A). Conversely, a low proportion (8.6–15.4%) were ‘dissatisfied’ or ‘very dissatisfied’ (Figure 5A). Similar results were obtained for questions about convenience, effectiveness and tolerability of the patch (data not shown). Most male participants who had been treated previously for OAB (54.7–60.6%) said that the patch offered significant or some benefits over previous treatments, suggesting that they preferred the patch over their prior therapy (Figure 5B). A small proportion preferred their previous medications (7.2–12.8%). Similar opinions of the patch vs. prior therapy were reported with respect to ease of use, effectiveness and tolerability (data not shown).

### Safety

Oxybutynin transdermal system was well tolerated; most men in the study (281/369; 76.2%) experienced no drug-related adverse event during up to 6 months of treatment. The most common type of treatment-related adverse event, application site reaction, occurred in 13.8% (51/369) of male participants (Table 2). The vast majority of these reactions (71/73; 97.3%) were judged mild or moderate in severity. Treatment-related anticholinergic adverse events were infrequently reported by male participants: dry mouth (*n* = 9; 2.4%), nausea (*n* = 5; 1.4%), headache (*n* = 4; 1.1%), constipation (*n* = 4; 1.1%), dizziness (*n* = 3; 0.8%), abdominal pain (*n* = 2; 0.5%), dry eye (*n* = 1; 0.3%) and dysuria (*n* = 1; 0.3%). The male participant who experienced treatment-related mild dysuria continued study treatment after a 2-day hiatus. A total of 47 men (12.7%) discontinued study participation because of a treatment-related adverse event. No male participants experienced a serious treatment-related adverse event.

**Table 2:** Treatment-related adverse events occurring in ≥ 1% of male participants

Adverse event	Male participants, *n* (%)
Application site reaction*	51 (13.8)
Dry mouth	9 (2.4)
Pruritus	8 (2.2)
Erythema	5 (1.4)
Nausea	5 (1.4)
Constipation	4 (1.1)
Headache	4 (1.1)
Rash	4 (1.1)
Somnolence	4 (1.1)

* A participant may have reported more than one type of application site (AS) reaction. Types included AS pruritus (22; 6.0%), AS dermatitis (20; 5.4%), AS erythema (15; 4.1%), AS irritation (6; 1.6%), AS pain (2; 0.5%), AS vesicles (2; 0.5%), AS bleeding (1; 0.3%), AS burning (1; 0.3%) and AS swelling (1; 0.3%).

Two participants (2/369; 0.5%) from the male cohort each reported one event of urinary retention, both of which were considered related to treatment. However, these events were mild in severity and neither required catheterisation. One participant who experienced urinary retention continued treatment with OXY-TDS, and the other discontinued treatment. Male and female study participants had comparable rates of urinary retention [2/369 men (0.5%) vs. 8/2508 women (0.3%)] and dysuria [1/369 men (0.3%) vs. 14/2508 women (0.6%)].

## Discussion

This planned subgroup analysis of MATRIX participants was among the longest-term, single, prospective assessments of the effectiveness of antimuscarinic treatment for OAB in a large cohort of men. As intended, the study population was representative of actual patients with OAB in terms of advanced age, comorbidity and use of concomitant medications. Many study participants had not persisted with previous OAB treatments, which also is a common characteristic of patients with OAB in the general population (12). MATRIX was the first study of men with OAB to have a patient-reported outcome (i.e. change in HRQoL) as its primary end-point. Concurrent use of the PPBC allowed comparison of participant-reported changes in global OAB severity, independent of the symptoms and HRQoL changes measured with the KHQ. This study did not have a placebo or active control; therefore, results cannot determine causal relationships. However, the open-label design was closer to clinical practice than blinded treatment. The large population size, minimally restrictive entry criteria and 6-month duration of the study also allowed better estimation of the incidence of relatively rare adverse events.

Global OAB severity in male participants, as self-assessed with the PPBC, improved distinctly from baseline to month 1 and continued to improve slightly during the remaining 5 months. Perceived urinary urgency (not enough ‘time to get to the bathroom’), which is a defining symptom of OAB, also improved markedly and within the same time as global OAB severity. This perceived change in urinary urgency is consistent with the significant (p < 0.001; kappa test of symmetry) improvement noted in the KHQ ‘urgency’ response item. Other symptoms and HRQoL items recorded with the KHQ also improved significantly during the study. These results are similar to those from a pooled analysis of two studies in men with OAB, in which treatment with TOL-ER significantly improved daytime and 24-h micturitions, although not nighttime micturitions (29). In MATRIX, treatment with OXY-TDS was associated with significant (p < 0.0001; kappa test of symmetry) improvement in urinary frequency and nocturia, as assessed through KHQ response items. The degree of HRQoL improvement in MATRIX was unaffected by pre-existing prostate conditions or the concomitant use of medications to treat BPH. These results are consistent with those of a randomised, controlled study of men with OAB and other LUTS, in which significantly more patients (172/215; 80%; p < 0.001) treated with TOL-ER and an α-blocker (tamsulosin) reported treatment benefit, compared with patients who received placebo (132/214; 62%) (15).

Depression symptoms monitored with the BDI-II decreased significantly during the study for men treated with OXY-TDS. The KHQ response item that asked participants whether they ‘Feel bad about yourself’ also showed significant (p < 0.05; kappa test of symmetry) improvement from baseline to study end. No other studies have shown improvement in depression among men being treated for OAB. Indeed, little evidence to date suggests that treatment improves depression symptoms in any population of patients with OAB, despite evidence that depression symptoms are common in these individuals (4). Kelleher et al. found no significant effect of TOL-ER or TOL-IR vs. placebo on the SF-36 Mental Summary score (7,30).

The number of male participants who were ‘satisfied’ or ‘very satisfied’ with OXY-TDS overall was four to eightfold greater, at various times during the study, than the number who reported being ‘dissatisfied’ or ‘very dissatisfied.’ For men with OAB who had been previously treated, the ratio of ‘satisfied/very satisfied’ to ‘dissatisfied/very dissatisfied’ also ranged from four- to eightfold at different times during the study. Taken together, these results suggest that perceived effectiveness, as well as convenience and perceived tolerability, was associated with treatment satisfaction in male participants.

Oxybutynin transdermal system was well tolerated by the male cohort in MATRIX and no serious adverse events were considered related to treatment. The rate of application site reactions in men (13.8%) was similar to that observed in the overall MATRIX population (14.0%) and in previous studies (17,18,20,22). A low incidence of anticholinergic adverse events was observed, despite the use of concomitant medications by the great majority of men. Despite some suggestion from another study that tolterodine and tamsulosin may have a synergistic effect on the incidence of dry mouth, in MATRIX, dry mouth did not occur significantly more frequently among men who took α-blockers than among those who did not (3/56 vs. 6/307; p = 0.142; Fisher's exact test) (15). No cases of acute urinary retention requiring catheterisation were reported in the male cohort described here, which was followed for up to 6 months. This low probability of urinary retention observed in individuals with BPH or other prostate problems is consistent with results from studies of TOL-ER treatment in patients with OAB, some of whom also had BOO or additional LUTS (15,29,31).

The International Continence Society definition of OAB is based on symptoms of urinary urgency, frequency and incontinence (32). Validated questionnaires that assess changes in these symptoms are important measures for improvement in OAB severity (33). The European Agency for the Evaluation of Medicinal Products has stated that ‘the primary aim for developing new drugs for UI should be to obtain a subjective improvement or cure of symptoms for the patient’ (34). In male study participants, perceived OAB severity, OAB symptoms and HRQoL all improved with OXY-TDS treatment. Serious adverse effects related to treatment were not observed during the study; urinary retention was rare (0.5% of all male participants) and resolved without the need for catheterisation. Satisfaction with treatment was good, even among those who had discontinued prior therapies. Participants in the present analysis were typical of OAB patients encountered in general practice in terms of their advanced age, multiple comorbid conditions and use of many concomitant medications. The use of questionnaires in general medical practice could allow effective treatment of many patients without the need for specialised urodynamic evaluations.

Although the design of MATRIX was appropriate for its goal of evaluating therapeutic success in a large, clinically relevant population, some inherent limitations should be noted. The study did not include objective assessments of response, such as urodynamic measurements or micturition frequency, but instead relied on participant-reported outcomes. It is not known how well symptoms recorded with the KHQ relate to symptoms measured with instruments that are commonly used to evaluate LUTS in men, such as the IPSS or the American Urological Association Symptom Score. However, evidence suggests a correlation between KHQ and IPSS results in Japanese patients (35,36). The *post hoc* analysis by history of prostate problems or concomitant BPH medication use was retrospective; therefore, selection bias may have influenced the results. Nonetheless, the positive outcomes reported in men who had a history of prostate problems or who were using BPH medications suggest that this last study limitation was of little consequence. In fact, a primary aim of MATRIX was to include individuals, such as these, who might be commonly encountered in clinical practice, but who have been excluded from nearly all randomised, controlled clinical trials conducted previously.

In summary, men with OAB reported improved OAB severity, HRQoL and depression symptoms after treatment with OXY-TDS. Treatment effects were similar in men with or without pre-existing prostate problems. Participant-reported perceptions of OXY-TDS supported the more objective measures of effectiveness and showed a high degree of satisfaction with treatment. OXY-TDS was well tolerated in men, none of whom experienced acute urinary retention or a treatment-related serious adverse event during up to 6 months of treatment. OXY-TDS appears to be a viable treatment for men with LUTS, many of whom have OAB alone or together with BPH/BPO (37).
